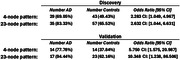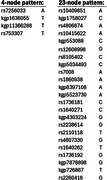# Identifying partial haplotypes that are associated with late‐onset Alzheimer disease

**DOI:** 10.1002/alz.093214

**Published:** 2025-01-03

**Authors:** Sharlee Climer

**Affiliations:** ^1^ University of Missouri ‐ St. Louis, Saint Louis, MO USA

## Abstract

**Background:**

Haplotypes are patterns of nucleotides in close proximity along a chromosome that are passed together across time and space. These patterns dictate the specific properties of proteins produced and the regulation of this production. General haplotype inference methods phase all provided genotypes within the region of interest into two haplotypes without regard for the ages or evolutionary impact of each mutation, thus force the inclusion of more recent and/or neutral mutations. Alternatively, BlocBuster (www.blocbuster.org) identifies partial haplotypes that include only highly correlated nucleotides using a unique correlation metric that anticipates heterogeneity of the individuals and an expanded network scaffolding. Given the heterogeneous nature of late‐onset Alzheimer disease (AD), we utilize BlocBuster to identify genetic risk patterns.

**Method:**

We analyzed WGS_Omni2.5M_20140220 data for chromosome 19 provided in the Alzheimer’s Disease Neuroimaging Initiative (ADNI) database(adni.loni.usc.edu). The data were arbitrarily split into 70% Discovery (42 AD, 69 MCI, 87 control) and 30% Validation (18 AD, 29 MCI, 37 control). BlocBuster builds a network model using AD, MCI, and control individuals (without labeling), where nodes represent SNP alleles and edges represent pairwise correlations between alleles, extracts clusters of inter‐correlated SNP alleles representing a partial haplotype, then tests the pattern for association with the given phenotype.

**Result:**

BlocBuster identified two patterns that are significantly associated with AD in both the Discovery and the independent Validation datasets(Table 1). The first represents a variant of the RUVBL2 gene and the second is a large allelic combination beginning upstream from DIRAS1 and spanning across SLC39A3, SGTA, and THOP1(Table 2).

**Conclusion:**

The network model was created blindly using all individuals in the Discovery dataset, yet two of the clusters were significantly associated with AD risk in both the Discovery and Validation data. Interestingly, THOP1 is a zinc‐activated oligopeptidase that is capable of cleaving beta‐amyloid precursor protein into amyloidogenic peptides. Furthermore, SLC39A3 is predicted to enable zinc transport across the membrane, SGTA is suggested to play a role in aggregate formation in neurodegenerative diseases, and methylation of DIRAS1 is associated with AD risk. These results identify interesting signatures that may pinpoint specific AD biological pathways, motivating further validation efforts.